# *Caenorhabditis elegans che-5* is allelic to *gcy-22*

**DOI:** 10.17912/micropub.biology.000313

**Published:** 2020-10-08

**Authors:** Hirofumi Kunitomo, Yuichi Iino

**Affiliations:** 1 Department of Biological Sciences, School of Science, The University of Tokyo

**Figure 1. che-5(e1073) carries mutations in gcy-22 that are responsible for chemotaxis defects of the mutants:**
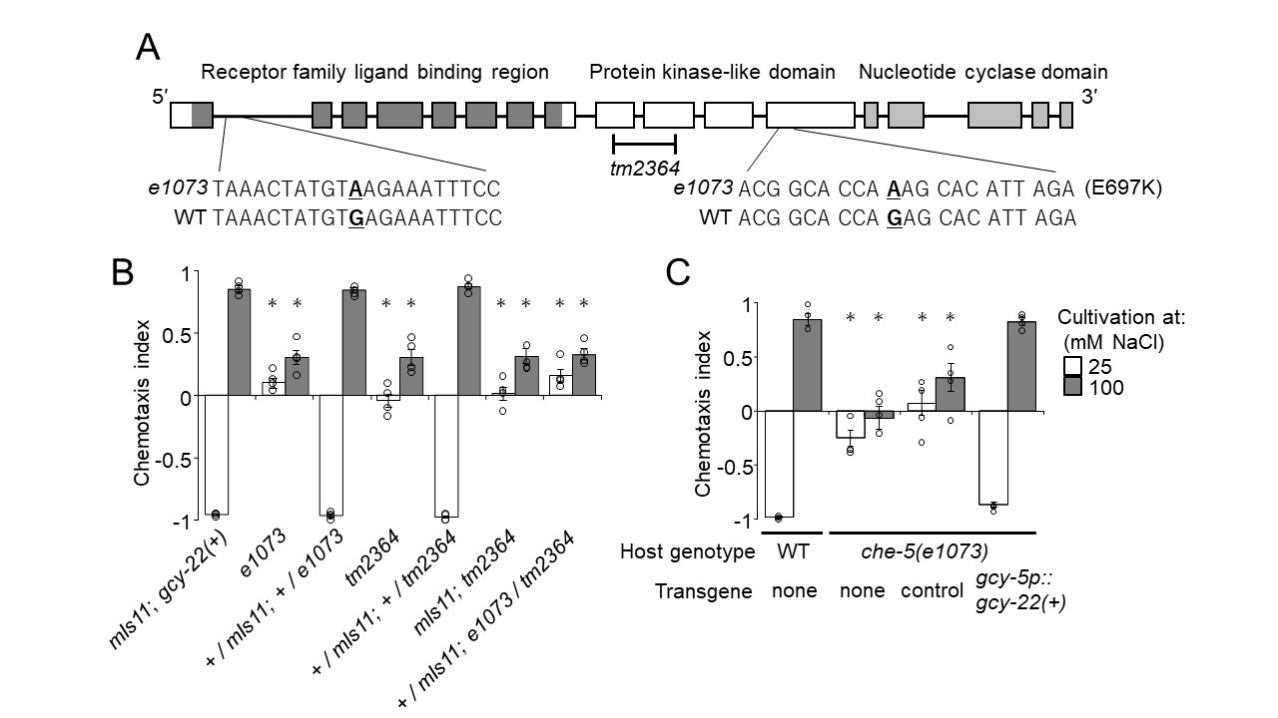
(A) Schematic diagram of *gcy-22a* gene structure. Boxes represent exons. Protein domains and the nucleotide substitutions found in *e1073* are indicated above and below the diagram, respectively. (B) Complementation test between *che-5(e1073)* and *gcy-22(tm2364)*. Salt concentration chemotaxis was observed in *e1073*/*tm2364* heterozygotes and their parental strains. *e1073* failed to complement *tm2364*. *n* = 5, Mean±SEM, *p ≤ 0.001, compared to *mIs11*, Dunnett’s test. (C) ASER-specific expression of *gcy-22(+)* rescued the chemotaxis defect of the *che-5(e1073)* mutants. *n* = 4 or 5, Mean±SEM, *p ≤ 0.01, compared to wild type, Dunnett’s test.

## Description

Mechanisms of chemotactic behaviors have been of great interest in *C. elegans* neuroscience since the early days of its research (Ward 1973). Lewis and Hodgkin (1977) systematically isolated more than ten abnormal chemotaxis (*che*) mutants that showed defective chemotaxis to sodium (Na^+^) and chloride (Cl^–^) ions (Lewis and Hodgkin 1977), whose responsible genes have already been molecularly characterized except for *che-5(e1073)*. We here show that *che-5(e1073)* is a missense allele of *gcy-22*, which encodes a receptor guanylyl cyclase (rGC) specifically expressed in the ASE-right (ASER) gustatory neuron and is essential for chemosensation through the neuron.

*C. elegans* is attracted to the NaCl concentration at which it has experienced with food, while avoid the concentration at which it has experienced starvation. ASER plays a major role in food-associated salt concentration chemotaxis; input of salt information into ASER is required and sufficient for chemotaxis to the salt concentration associated with food (Kunitomo *et al.* 2013). ASE neurons, consisting of bilaterally symmetrical ASE-left (ASEL) and ASER, are the major sensory neuron for water-soluble attractants (Bargmann and Horvitz 1991). They respectively sense different sets of ions such as Na^+^ and Cl^–^ (Pierce-Shimomura *et al.* 2001; Suzuki *et al.* 2008; Ortiz *et al.* 2009). A cyclic GMP (cGMP) signaling pathway consisting of rGCs and TAX-4/TAX-2 cyclic nucleotide-gated ion channels mediates sensory transduction in ASE (Coburn and Bargmann 1996; Komatsu *et al.* 1996; Ortiz *et al.* 2009). ASEL and ASER express distinct sets of rGCs (Ortiz *et al.* 2006). Of these, *gcy-22* is essential for ASER to respond to multiple ion species; therefore it is proposed as a common component of chemoreceptor complexes (Ortiz *et al.* 2009; Adachi *et al.* 2010; Smith *et al.* 2013). To further elucidate the mechanisms of chemosensation through ASER, we characterized as yet uncloned *che-5*. We focused on *che-5* because CB1073 *che-5(e1073)* mutant, the unique strain/allele of the gene, showed chemotaxis defects characteristic of ASER-specific malfunction; a severe defect in attraction to Cl^–^, whereas relatively moderate defect in Na^+^ chemotaxis (Lewis and Hodgkin 1977).

We mapped the mutation of *che-5(e1073)* responsible for salt chemotaxis defect between genetic positions 24.52 (single nucleotide polymorphism (SNP): WBVar00240760) and 25.54 (SNP: WBVar00053592) on chromosome V by using SNPs between N2 and CB4856 (Wicks *et al.* 2001). The mapped region contained an ASER-specific chemotaxis gene, *gcy-22* (genetic position: 25.28). This result was unexpected from the initial report that mapped *e1073* on chromosome IV (Lewis and Hodgkin1977), but consistent with a recent observation in which whole-genome sequencing failed to identify a mutation corresponding to *che-5(e1073)* on chromosome IV (Smith *et al.* 2013).

*gcy-22(tm2364)* harbors a deletion in the middle of *gcy-22* coding region that results in a frame shift and therefore is a putative null allele (Fig. 1A). Salt concentration chemotaxis of the animals heterozygous for *e1073* and *tm2364* showed that the two alleles failed to complement each other, indicating that these alleles affect the same locus (Fig. 1B). Nucleotide sequencing of the *gcy-22* locus revealed that *e1073* carried ACG GCA CCA **A**AG CAC ATT AGA in which the adenine residue in bold letter was guanine in wild type (Fig. 1A). This transition results in a missense change E697K in GCY-22 isoform a. The glutamate residue is located in the kinase-like domain and well conserved in rGC proteins. In addition, CB1073 carried another nucleotide substitution within the first intron of *gcy-22*, TAAACTATGT**A**AGAAATTTCC, in which the adenine residue in bold letter was guanine in wild type (Fig. 1A). Furthermore, expression of a cDNA for *gcy-22a* in CB1073 in ASER-specific manner completely rescued the salt chemotaxis defect of the mutant (Fig. 1C). These results strongly indicate that *che-5* is allelic to *gcy-22* and the chemotaxis defect of *e1073* is due to the mutations of *gcy-22* locus.

## Methods

Salt concentration chemotaxis was evaluated as described (Kunitomo *et al.* 2013). A chemotaxis index was calculated to quantify the behavior as follows. Chemotaxis index = {(*N* at high NaCl-region) – (*N* at low NaCl-region)} / {(total *N*) – (*N* that did not move from the origin)}, in which *N* represents number of animals. For complementation tests, males of PD4792 (*mIs11* with *gcy-22(+)* background) or JN2608 (*mIs11* with *gcy-22(tm2364)* background) were mated with CB1073 hermaphrodites. F1 progenies were tested for salt concentration chemotaxis, and crossed progeny hermaphrodites that carried *mIs11* were separately counted from self-progeny hermaphrodites that did not carry the marker. For rescue experiments, 5 ng/microL *gcy-5p::gcy-22a* construct was introduced into CB1073 with 15 ng/microL *myo-3p::venus* as a transformation marker.

## Reagents

Strains. The JN strains are available upon request. Others are available at Caenorhabditis Genetic Center (CGC).

Bristol N2: wild type

CB4856: wild type

CB1073: *che-5(e1073)* V.

JN967: *gcy-22(tm2364)* V.

JN2606: *che-5(e1073)* V; *peEx2606[myo-3p::venus]*.

JN2607: *che-5(e1073)* V; *peEx2607[gcy-5p::gcy-22a myo-3p::venus]*.

JN2608: *mIs11[myo-2p::GFP pes-10p::GFP gut-promoter::GFP]* IV; *gcy-22(tm2364)* V.

PD4792: *mIs11[myo-2p::GFP pes-10p::GFP gut-promoter::GFP]* IV.
